# Young Adults On Mental Health Instagram and TikTok: Self-Care, Self-Diagnosis and Performances of The Mentally Healthy Self

**DOI:** 10.1192/j.eurpsy.2024.130

**Published:** 2024-08-27

**Authors:** A. Bailie

**Affiliations:** Politics and International Relations, University of York, York, United Kingdom

## Abstract

**Abstract:**

My PhD research critically examines the contemporary U.K politics of mental health and Illness and mental illness amongst young adults via social media.

This presentation examines the way in which social media, like Instagram and Tiktok allows young adults to explore, express and share their selfhood and identity around ideas of mental health and illness through videos, posts and online interactions. I will briefly explore how young adults use social media content to learn, experience and criticise their lived experiences, care for and treatment of mental health and illness. Arguing that these new developments in language and social practices around mental health and illness via social media need to be further explored, acknowledged and addressed in social science and this can be supported by work in the field of psychiatry.

To illustrate this, I will share empirical data from interviews conducted in 2021 and 2022 with young adults who speak about their mental health and illness online and have engaged with psychiatric services, for example, in-patient settings. Instagram and Tiktok have become important arenas for young adults because of the informative role they play in young adults’ understanding of what a healthy person looks like, or their ideas of *the mentally healthy self.* A concept I introduce in my research of which I will explain and explore through the presentation. In young adults’ understandings of mental health and illness there is a movement towards social practices that are intended to achieve (an idea of ) mental health and this is created in a powerful digital environment that is affected by capitalism, neoliberal discourse and exists in a context of multiple political and health crises in the U.K and globally. Overall, the presentation argues that these mental health and illness social practices have transformed young adults’s experiences by responsibilisation in self-care that both isolates and empowers their experiences.

**Disclosure of Interest:**

None Declared

